# A Two-Stage Adaptive Laboratory Evolution Strategy to Enhance Docosahexaenoic Acid Synthesis in Oleaginous Thraustochytrid

**DOI:** 10.3389/fnut.2021.795491

**Published:** 2021-12-31

**Authors:** Sen Wang, Weijian Wan, Zhuojun Wang, Huidan Zhang, Huan Liu, K. K. I. U. Arunakumara, Qiu Cui, Xiaojin Song

**Affiliations:** ^1^Key Laboratory of Biofuels, Qingdao Institute of Bioenergy and Bioprocess Technology, Chinese Academy of Sciences, Qingdao, China; ^2^Shandong Provincial Key Laboratory of Energy Genetics, Qingdao Institute of Bioenergy and Bioprocess Technology, Chinese Academy of Sciences, Qingdao, China; ^3^Shandong Energy Institute, Qingdao, China; ^4^Qingdao New Energy Shandong Laboratory, Qingdao, China; ^5^University of Chinese Academy of Sciences, Beijing, China; ^6^Department of Crop Science, Faculty of Agriculture, University of Ruhuna, Kamburupitiya, Sri Lanka; ^7^Center for Ocean Mega-Science, Chinese Academy of Sciences, Qingdao, China; ^8^Shandong Engineering Laboratory of Single Cell Oil, Qingdao Institute of Bioenergy and Bioprocess Technology, Chinese Academy of Sciences, Qingdao, China; ^9^Qingdao Engineering Laboratory of Single Cell Oil, Qingdao Institute of Bioenergy and Bioprocess Technology, Chinese Academy of Sciences, Qingdao, China

**Keywords:** adaptive laboratory evolution, thraustochytrid *Aurantiochytrium*, docosahexaenoic acid, heavy-ion irradiation, lipid accumulation

## Abstract

Thraustochytrid is a promising algal oil resource with the potential to meet the demand for docosahexaenoic acid (DHA). However, oils with high DHA content produced by genetic modified thraustochytrids are not accepted by the food and pharmaceutical industries in many countries. Therefore, in order to obtain non-transgenic strains with high DHA content, a two-stage adaptive laboratory evolution (ALE) strategy was applied to the thraustochytrid *Aurantiochytrium* sp. Heavy-ion irradiation technique was first used before the ALE to increase the genetic diversity of strains, and then two-step ALE: low temperature based ALE and ACCase inhibitor quizalofop-p-ethyl based ALE were employed in enhancing the DHA production. Using this strategy, the end-point strain E-81 with a DHA content 51% higher than that of the parental strain was obtained. The performance of E-81 strain was further analyzed by component analysis and quantitative real-time PCR. The results showed that the enhanced in lipid content was due to the up-regulated expression of key enzymes in lipid accumulation, while the increase in DHA content was due to the increased transcriptional levels of polyunsaturated fatty acid synthase. This study demonstrated a non-genetic approach to enhance lipid and DHA content in non-model industrial oleaginous strains.

## Introduction

Very long-chain polyunsaturated fatty acids (ω-3) (ω-3 PUFAs), such as eicosapentaenoic acid (EPA, C20:5) and docosahexaenoic acid (DHA, C22:6), are considered as the essential fatty acids in human nutrition and health (1–3). Since human are not able to synthesize EPA and DHA *de novo*, therefore, adequate intakes from external sources are required (4). Currently, deep-sea fish oil is the traditional source of PUFAs, while it is insufficient to meet the global demand for PUFAs (5–7). Thraustochytrid *Aurantiochytrium*, a heterotrophic non-photosynthetic protist, is well-known for its capacity to accumulate DHA (8). Its biomass accumulation could be more than 100 g/L, with the total lipid over 40% of dry cell weight (DCW) and DHA content over 40% of total fatty acids (TFAs) (9–11). Moreover, thraustochytrids can use glucose, glycerol, and molasses, etc., as a carbon source for fermentation, while some species have the xylose utilization capacity (11–13). The broad substrate utilization capacity increases the potential of thraustochytrids as microbial cell factories for lipid biosynthesis. Thus, oil produced from *Aurantiochytrium* appears to be a sustainable resource to fill the gap between the demand and supply of DHA (3, 14). However, its commercial exploitation has been restricted by the substandard productivity and high fermentation costs. Currently, several genetic engineering strategies have been successfully performed to optimize the lipid-accumulating capacity of *Aurantiochytrium* (15–18). However, the use of genetic modified strains is prohibited in food industry in many countries, and consumer acceptance remains a contentious issue (19). Therefore, an adaptive laboratory evolution (ALE) strategy has been developed in order to obtain non-transgenic strains with high DHA content.

Microorganisms are capable of acquiring beneficial phenotypes through random genetic mutations, thus they can rapidly adapt to changing environments. During the ALE process, microorganisms are repeatedly grown under certain stress conditions to induce positive phenotypes. In contrast to genetic modified engineering, ALE enjoys the advantage of regulating many different genes in parallel without the introduction of other genes (20). ALE has been successfully applied in strains improvement, including important model organisms, such as *Saccharomyces cerevisiae* (21), and many microalgae, such as *Crypthecodinium cohnii* (22) and *Dunaliella salina* (23). In recent years, ALE was also applied in thraustochytrids to modify strains. Sun et al. developed a high-oxygen based ALE strategy in thraustochytrid *Schizochytrium* to improve its growth performance (24), a high salinity based ALE method to improve its lipid production (20), and a cooperative two-factor ALE method to enhance both the final biomass and lipid content (25). All these studies demonstrate ALE is a powerful method to enhance the specific properties of thraustochytrids, however, more innovative selective pressures still need to be identified and applied to improve the DHA content.

Although compared with genetic engineering, ALE has some major benefits, it also has some inherent limitations such as the longer running time and the higher operating cost. Mutations are considered as a basis of ALE, and the increase of genotypic diversity can speed up evolution process (21). In a *Escherichia coli* study, a combined ALE with genome shuffling strategy successfully enhanced the desired n-butanol tolerance (26). Similarly, the multiplexed automated genome engineering (MAGE) technology was applied to expedite the design and evolution of organisms with new and improved properties (27). Thus, increasing mutation rate may expedite the evolutionary process. Considering the application area of targeted strains, non-genetic modified methods may be more suitable for the food industry. Heavy-ion irradiation is a novel and powerful mutagenic technique that is capable of inducing a broad range of mutations. Due to the higher linear energy transfer (LET), it possess the ability to break DNA double-strand more effectively than the other mutagenic methods; such as ethyl methane sulfonate (EMS), X-rays or γ-rays (28). Therefore, heavy-ion irradiation was applied before ALE to improve the diversity of starting strains.

Since inhibiting enzyme proteins can perturb or even inhibit metabolism, thus the selective pressure of enzyme inhibitors can also be applied in ALE process to improve the characteristics of organisms. Recently, an ACCase inhibitor based ALE was successfully applied in *C. cohnii* to improve the lipid accumulation (29). However, the enzyme inhibitors usually have a significant negative effect on biomass accumulation. For example, although the tested enzyme inhibitors could improve lipid productivity, they all inhibit the cell growth in *Chlamydomonas reinhardtii* at varying degrees (30). In any case, enhancing lipid biosynthesis by adding the enzyme inhibitors in the ALE process may be a useful strategy.

In this study, a non-genetic modified approach was performed in non-model oleaginous thraustochytrid *Aurantiochytrium* to obtain a mutant with high DHA yield though a two-stage ALE strategy. We first applied heavy-ion irradiation technique to increase the genotypic diversity of strain, and then a two-step ALE was used to enhance the DHA content. Finally, a E-81 strain was got with the DHA production increases by 51% compared with that in starting strain. This study demonstrated a non-genetic approach to efficiently enhance lipid and DHA content in non-model industrial oleaginous strains.

## Materials and Methods

### Strains and Culture

*Aurantiochytrium* sp. SD116 was isolated from the mangrove and reported in our previous study (8). It was cultured in seed liquid medium containing 30 g/L glucose, 10 g/L yeast extract, and 10 g/L artificial sea salt. The culture was shaken at 200 rpm and grown at 25°C. *Aurantiochytrium* cells were then transferred into fermentation medium containing 60 g/L glucose, 20 g/L yeast extract, and 15 g/L sea salt to determine the lipid profiles.

### Heavy-Ion Irradiation Mutagenesis

*Aurantiochytrium* cells at logarithmic phase were subjected to heavy-ions irradiation mutagenesis. The cells were exposed to an ion beam at the Heavy-Ion Research Facility in Lanzhou, Institute of Modern Physics, Chinese Academy of Sciences where the irradiation was done with different doses (0, 20, 40, 80, 120, 160, and 200 Cy) of carbon ions (^12^C^6+^). The carbon ion energy was measured as 80 MeV/u, and the average linear energy transfer (LET) value was 31 keV μm^−1^. After irradiation, the cells were placed on seed medium plates to assess the mortality. Cell survival rate = colony counts of irradiated cells/colony counts of irradiated cells with 0 Gy × 100%. And cell mortality = 100% – cell survival rate.

### Adaptive Laboratory Evolution (ALE)

**Figure 1:**
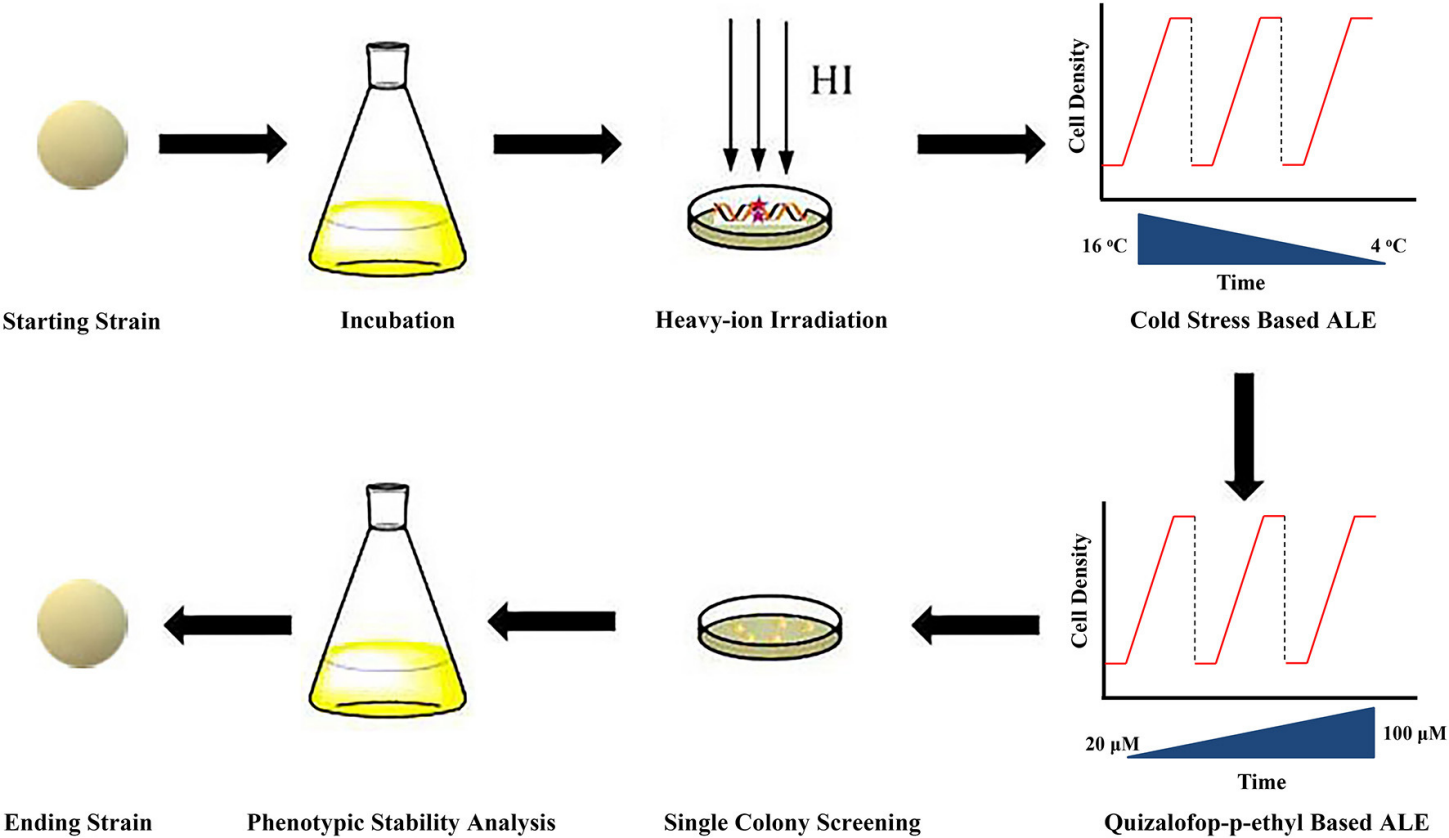
Overview of the modified ALE strategy. HI, heavy-ion irradiation; CS, cold stress; QE, quizalofop-p-ethyl.

Cells were inoculated into the seed medium where the ALE process commenced. Then the temperature of the medium was gradually decreased from 16 to 4°C with 4°C per change (Figure 1). When the cells entered into stationary phase (biomass was almost no changed after 12 h), an aliquot of 2% (v/v) culture was re-inoculated into a fresh medium. During the evolution process, the growth rate of the cells was found to be increased. When the growth rate reached the maximum, the temperature of the medium was gradually decreased. This experimental evolution proceeded for ~20 cycles for ~100 days, and the endpoint strains were named as E-C strains.

Before the second-round ALE, the cell mortality was firstly assessed with different concentrations (0, 5, 10, 15, 20, 25, and 30 μM) of quizalofop-p-ethyl in the agar plates containing seed medium. Cell survival rate = colony counts with different doses of quizalofop-p-ethyl/colony counts with 0 μM quizalofop-p-ethyl × 100%. And cell mortality = 100% – cell survival rate. Then the ACCase inhibitor quizalofop-p-ethyl was used for the second-round ALE. The concentration of quizalofop-p-ethyl was gradually increased from 20 to 100 μM with 20 μM per change. After the growth rate reached the maximum at 100 μM quizalofop-p-ethyl, the evolution proceeded for 10 cycles for ~60 days. Then the endpoint strains were plated on seed medium plates to select the single colonies.

### Quantitative Real-Time PCR (qRT-PCR) Analysis

Briefly, cells of *Aurantiochytrium* were harvested and immersed in RNAlock Reagent (TIANGEN, China), and stored at −80°C until use. Total RNA was isolated using TRIzol reagent (Invitrogen, USA) according to the manufacturer's instructions. RNA purity was checked using a Nano-300 spectrophotometer (Aosheng, China), and RNA degradation and contamination was monitored on 1% agarose gels. Synthesis of cDNA was carried out with a Revert Aid First strand cDNA Synthesis Kit (Thermo Scientific), and cDNA was used as the template for qRT-PCR analysis with the primers listed in Supplementary Table 1. Actin was used as an internal control to normalize the expression levels. Then, the relative abundance of different mRNA molecules was calculated based on the previous method (29).

### Biomass, Glucose Consumption, Lipid, Fatty Acid Composition Analysis, and Component Analysis

Biomass was expressed as dry cell weight (DCW). Five milliliters samples were harvested and freeze-dried to constant weight at −50°C. The glucose concentration was analyzed with a SBA-40E Biosensor (Institute of Biology, Shandong Academy of Sciences, China).

Total lipid was extracted using a combination solvent of chloroform and methanol (2:1, v/v). The extracted lipids were transferred to a pre-weighed glass tube and vacuum evaporated under 50°C, then the total lipid was weighed. To obtain the fatty acid methyl esters (FAMEs), the total lipid was dissolved in 1 mL of chloroform and incubated with 2% (v/v) sulfuric acid/methanol at 85°C for 2.5 h. FAMEs were determined by gas chromatography (Agilent Technologies, 7890B) (31).

Kjeldahl nitrogen determination method was employed in determining the protein content (32). Lyophilized samples were hydrolyzed with 6 M hydrochloric acid for 20 h, and then amino acids were measured by the amino acid analyzer (A300; membraPure, Germany).

The carbohydrate content was assayed following the Phenol-Sulfuric acid method. Lyophilized samples were ground using a mortar and then hydrolyzed in boiling water with 6 M hydrochloric acid for 0.5 h. After cooling, 10% (w/w) NaOH was used to regulate pH = 7.0. Hydrolysate was then taken for carbohydrate content determination by the Phenol-Sulfuric acid method (33).

### Fed-Batch Fermentation

The fed-batch fermentation experiment was performed in a 5 L Biostat® B plus bioreactor as described previously (34). The cultures were grown in 2.5 L of initial fed-batch fermentation medium at 25°C. The aeration and stirring speed are fixed at 2 VVM and 800 rpm, respectively. The glucose concentration was estimated with the SBA-40E Biosensor and maintained at about 20 g/L by continuously feeding of a supplement with a glucose concentration of 800 g/L. Moreover, 100 mL of yeast extract solution with a concentration of 150 g/L was added every 24 h, until 72 h of fermentation. The pH was maintained at 6.5 by adding 2 M NaOH. The initial fed-batch fermentation medium contained 100 g/L glucose, 10 g/L yeast extract, 5 g/L tryptone, 5 g/L KH_2_PO_4_, 1 g/L MgSO_4_, and 15 g/L artificial seawater. One milliliter of antifoam, THI®X-298 (Thinking Finechem, Yantai, China), was added at the beginning to control foam formation. Samples (50 mL) for off-line determination of biomass, glucose, lipid, and fatty acid profiles were drawn at 12 h intervals until the end of the fermentation.

### Calculation and Statistical Analysis

All data are the means of three replicates and reported as the mean ± SE. The statistical analysis was carried out by Excel, and the significance of differences (*p* < 0.05 and *p* < 0.01) was assessed using a *t*-test.

## Results and Discussion

### Enhancement of Diversity of Starting Strains via Heavy-Ion Irradiation

Mutations are the basis underlying ALE, thus increased mutation rate could expedite the evolutionary process (27). Heavy-ion irradiation is a powerful mutagenic technique that is capable of inducing a broad range of mutations. In the present study, heavy-ion irradiation was applied before ALE to increase the genetic diversity of starting strains. A higher mutation rate can be effective to a certain extent only, because it may also lead a genetic burden. Therefore, the effect of the dosage of irradiation on cell mortality was first investigated. When *Aurantiochytrium* cells were exposed to seven heavy-ion irradiation doses (0, 20, 40, 80, 120, 160, and 200 Gy), a dose-dependent mortality was observed (Supplementary Figure 1). The mortality rate was found to be increased approximately from 50 to 80%, respectively, for 120 and 160 Gy, which were regarded as the best range for mutation breeding. Therefore, mutants from 120 and 160 Gy irradiation treatments were selected for ALE experiment.

### High DHA Production Strain E-81 Obtained From ALE

It is known that PUFAs play an important role in resisting stresses such as low temperature by increasing the fluidity of microbial cell membranes (35, 36). According to previous reports (37, 38), low temperatures can promote the biosynthesis of PUFAs in thraustochytrids while decrease their growth rate. Therefore, the first-round ALE aiming at enhancing the DHA content was conducted under cold stress conditions. Mutant cells generated through the irradiation treatments (120 and 160 Gy) were used as the starting strains, which were subjected to the ALE. The temperature was decreased from 16 to 4°C during the entire ALE process (Figure 1). Finally, the strains with the maximum growth rate at 4°C were named as E-C strains. The content of DHA in E-C strains was found to be increased by 15% compared to that of SD116, which is 48% of its total fatty acids (TFA) (Supplementary Figure 2). However, in terms of the total lipid content, both E-C and SD116 strains were found to be almost alike (Supplementary Figure 2).

Acetyl-CoA carboxylase (ACCase) is capable of catalyzing the first bottleneck step in fatty acid biosynthesis. As reported in previous studies, the lipid production in oleaginous microorganisms could be increased by the enhanced activity of ACCase (15, 39). Quizalofop-p-ethyl is an ACCase inhibitor that usually used to enhance lipid accumulation in organisms. Chaturvedi screened *Nannochloropsis oculata* mutants with quizalofop-p-ethyl and found that the ACCase activity of herbicide-resistance microalgae had increased ~2-fold (40). Another study reported that the lipid content of mutagenized microalgae was 59% higher than wild type, indicating that screening mutants with the herbicide quizalofop-p-ethyl could lead to enhance lipid accumulation (41). Therefore, quizalofop-p-ethyl was selected in the second-round of ALE. As shown in Supplementary Figure 3, the cell mortality rate at 30 μM quizalofop-p-ethyl was almost 100%. Therefore, 20 μM quizalofop-p-ethyl was used as the started concentration for ALE process. Unlike the result of cell mortality rate, cells could grow in the lipid medium with 30 μM quizalofop-p-ethyl. The concentration of quizalofop-p-ethyl was thus increased gradually from 20 to 100 μM during the process. Similar to the cold stress-based ALE, the cell growth rate reached the maximum at 100 μM quizalofop-p-ethyl, and then the streak plate method was performed to select the single colonies.

**Figure 2:**
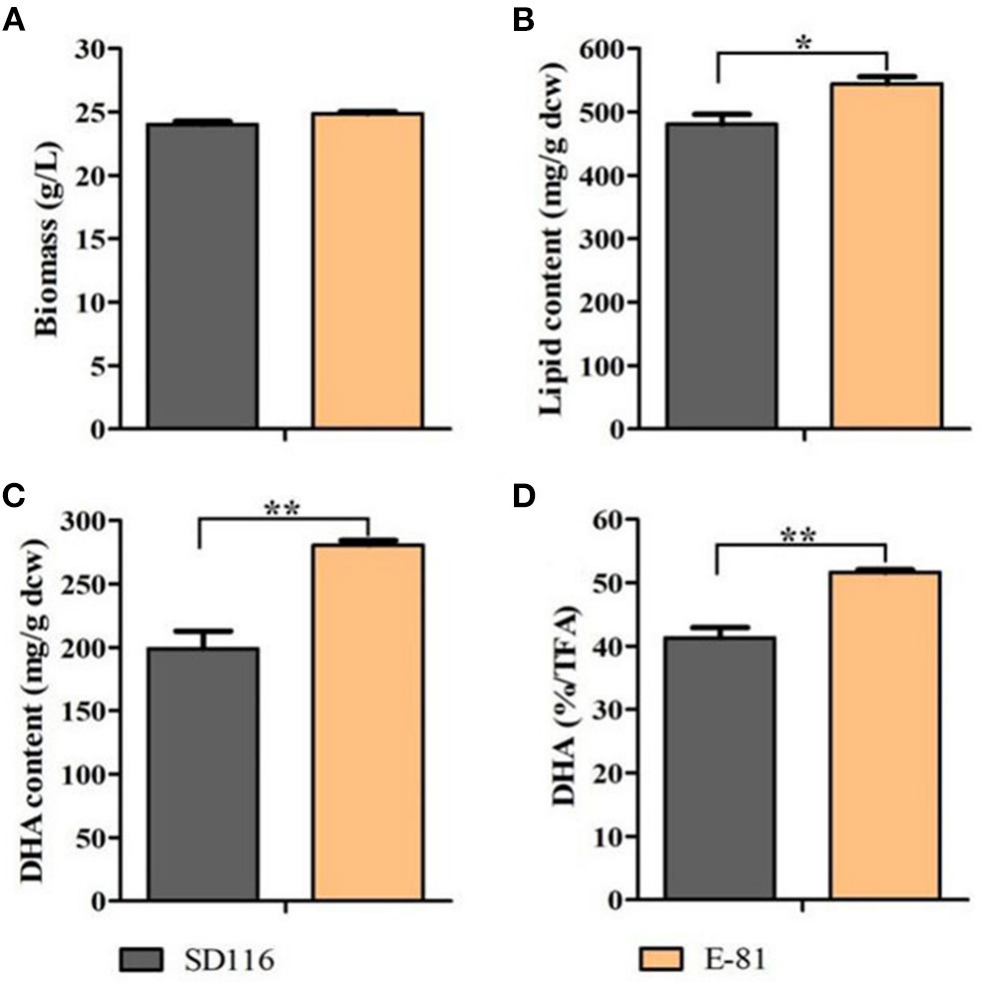
Analysis of the biomass and lipid profiles in SD116 and E-81. **(A)** Biomass, **(B)** lipid content, **(C)** DHA content, and **(D)** DHA purity in total fatty acids (TFA). ^**^P < 0.01 and ^*^P < 0.05.

96 single colonies were taken and then cultured in fermentation medium for 96 h. Lipid profiles of these strains were screened (Supplementary Figure 4) to select the strain with the highest DHA yield and purity. As shown in Figure 2, the biomass yield of E-81 was equivalent to that of SD116. And the total lipid content had reached 544 mg/g DCW, which was 13% higher than SD116 (Figure 2B). In addition, the DHA yield and purity in E-81 strain were increased by 41 and 25%, respectively, compared to those of SD116 (Figures 2C,D).

All inclusive, a modified ALE strategy was designed for *Aurantiochytrium* sp. SD116 to enhance the DHA purity and yield (Figure 1): (1) heavy-ion irradiation strategy was used to increase the diversity of starting strains; (2) cold stress based ALE and quizalofop-p-ethyl based ALE were performed to improve the PUFA content and total lipid production, respectively; and finally, (3) the phenotypic stable ending strain with high DHA yield and purity was obtained by continuously passaging.

### Changes of Metabolic Network in Strain E-81

**Figure 3:**
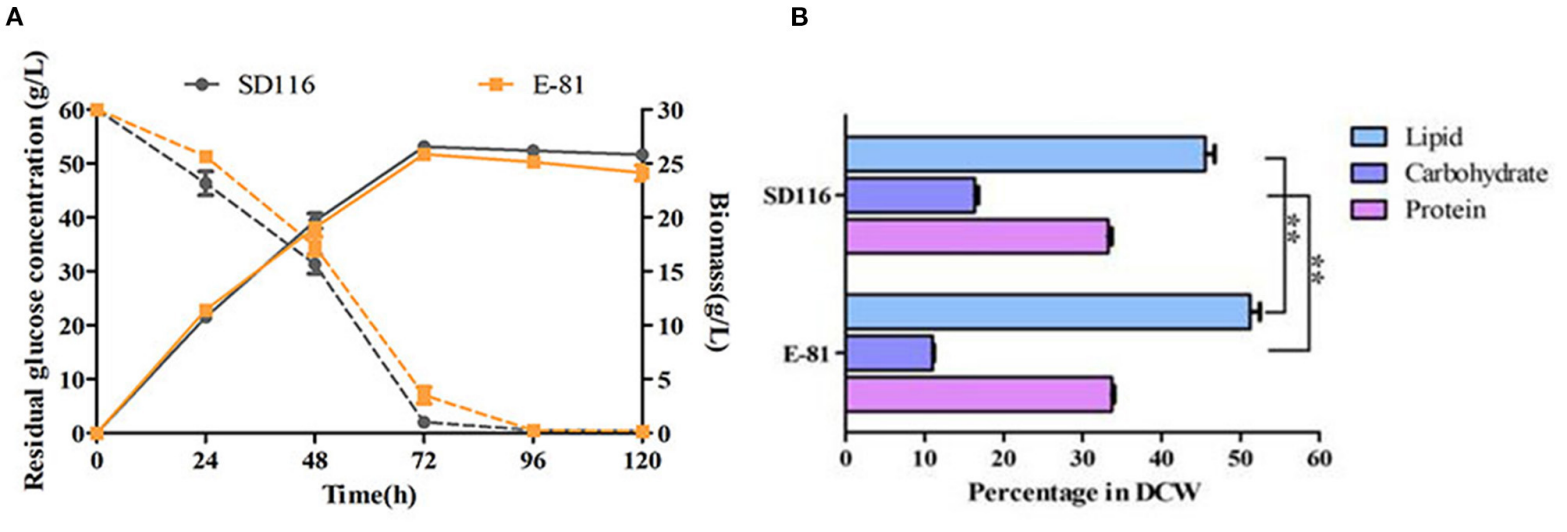
Characterization of cell growth **(A)** and biomass composition **(B)** in strains SD116 and E-81. ^**^P < 0.01.

As shown in Figure 3A, both the cell growth and glucose consumption rates of E-81 were similar to those of SD116, implying that the biomass productivity in E-81 was almost comparable to that of SD116. Taking the lipid content of E-81, which showed a significant increase (~55% of DCW) into account, we hypothesized that E-81 has the potential to convert more carbon to lipid by rewiring the intracellular metabolism. As shown in Figure 3B, the protein contents of SD116 and E-81 were indistinguishable, though, a noticeable decrease in total carbohydrate content was observed in E-81 compared to that of in SD116 (~5% of DCW). These results implied that E-81 strain could redirect the carbon allocation from carbohydrate to lipid. To verify this hypothesis, the transcriptional levels of key enzymes in lipid accumulation were analyzed.

**Figure 4:**
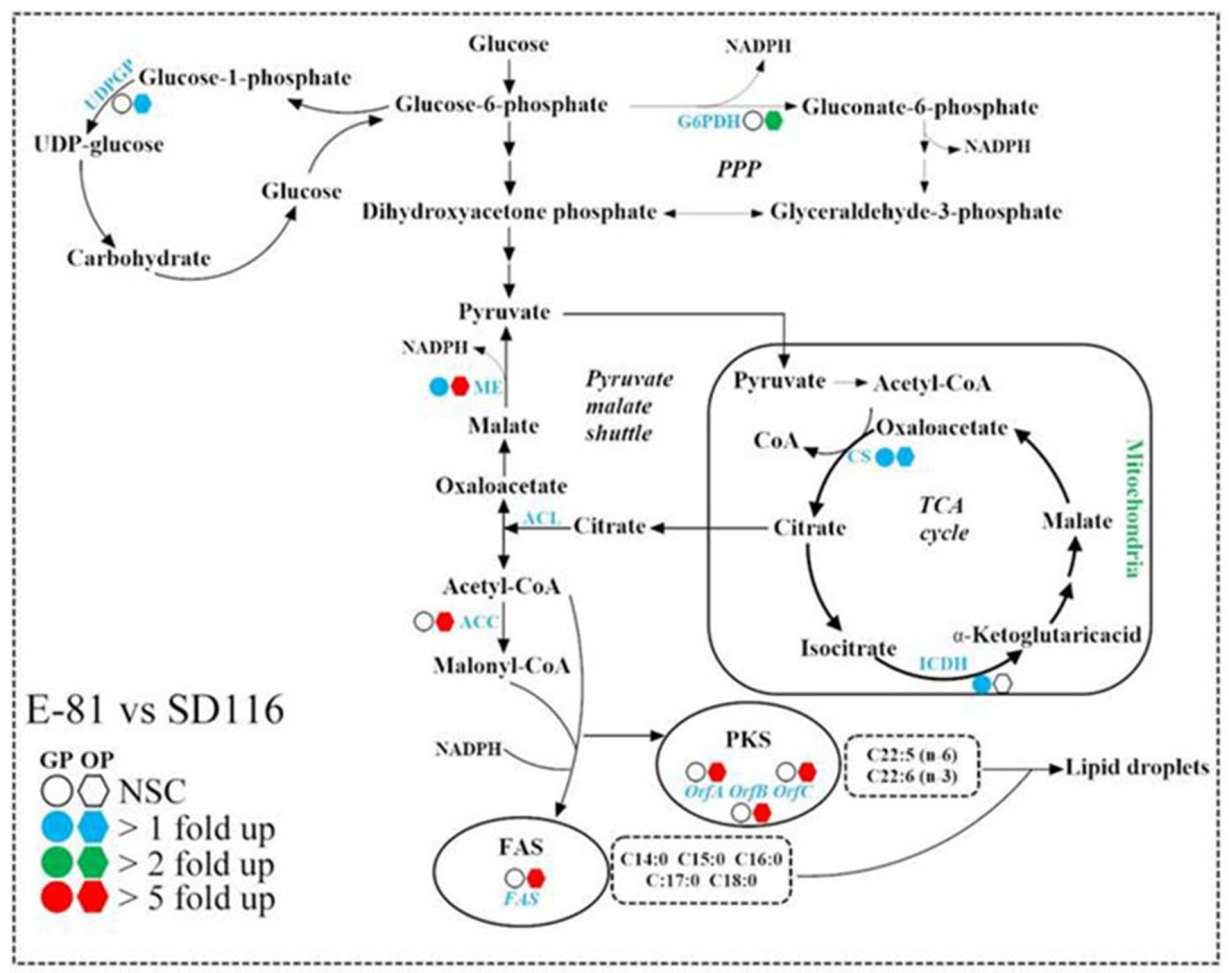
Comparison of the transcription levels of key enzymes in fatty acid synthesis pathways of SD116 and E-81 strain. G6PDH, glucose-6-phosphate dehydrogenase; ME, malic enzyme; CS, citrate synthase; ICDH, isocitrate dehydrogenase; ACL, ATP citrate lyase; ACC, acetyl-CoA carboxylase; FAS, fatty acid synthase; PKS, polyketide-like polyunsaturated fatty acid synthase; OrfA, PKS subunit A; OrfB, PKS subunit B; OrfC, PKS subunit C; UDPGP, UDP-glucose pyrophosphorylase; NSC, no significant change; GP, growth phase; OP, oleaginous phase.

The growth cycle of *Aurantiochytrium* sp. is characterized by two distinct physiological stages, namely the growth phase and the oleaginous phase. Therefore, the transcription profiles at 48 and 72 h which represented these two phases, respectively, were monitored. During the growth phase, the oleaginous microorganisms convert the carbon source into cell mass, which is rich in proteins, but poor in quantities of lipids (42). As shown in Figure 4, the transcription levels of the genes responsible for fatty acid biosynthesis which include *FAS, OrfA, OrfB*, and *OrfC* (43) were not significantly varied between E-81 and SD116 in the growth phase, though the transcription levels of citrate synthase (*CS*), isocitrate dehydrogenase (*ICDH*), and malic enzyme (*ME*) were significantly increased in E-81 strain than the other. *CS* and *ICDH* are the key enzymes in TCA cycle, whereas ME is the key enzyme in pyruvate malate shuttle. Both of these pathways are known to generate biosynthetic precursors which are involved in the production of energy or reducing power that are essential for the biosynthesis of various macromolecules. Therefore, the increased transcription levels of *CS, ICDH*, and *ME* in E-81 strain may provide more biosynthetic precursors.

Growth phase is followed by the lipid accumulation in the oleaginous phase. ICDH inhibition is critical at the beginning of lipogenesis, because the disturbance of the TCA cycle could induce an intra-mitochondrial accumulation of citric acid which is then excreted to the cytoplasm in exchange with malate (42). At the oleaginous phase of the present study, the transcription levels of *CS* and *ME* were up-regulated while no changes of the transcription level of *ICDH* was observed in E-81 strain, suggesting that more acetyl-CoA could be synthesized in E-81 strain than SD116. The accumulated pool of acetyl-CoA along with reducing power could increase lipid and DHA productivities (44). In the oleaginous phase, the transcription level of *ACC* in E-81 strain increases more than 5-fold compared with that in SD116, indicating ACCase inhibitor based ALE is an effective approach to increase ACCase expression, and more carbon resources can be used for lipid biosynthesis. There are two competing fatty acid synthesis pathways in *Aurantiochytrium* [(17); Figure 4]. Fatty acid synthase (FAS) pathway is mainly responsible for synthesis of saturated fatty acids (SFA), and polyketide synthase-like fatty acid (PKS) pathway which contains three genes namely, *OrfA, OrfB*, and *OrfC* encoding for PKS proteins synthesizes PUFA. The ratio of the transcription levels of PKS and FAS is closely related to the fatty acid composition. Compared with SD116, all the genes involved in lipid synthesis, including fatty acid biosynthesis genes *FAS, OrfA, OrfB*, and *OrfC*, and NADPH biosynthesis genes *ME* and *G6PDH*, were significantly up-regulated in strain E-81. This may be attributed to the increased total lipid production in strain E-81. Furthermore, the ratio of the transcription levels of PKS and FAS were more evidently up-regulated in E-81 strain than in SD116 at the oleaginous phase (Supplementary Figure 5), which explained the reason for higher PUFA content in E-81 strain.

Previous studies have showed that some microalgae could increase lipid productivity by inhibiting the biosynthesis of protein or starch (29, 45, 46). Taking the complexity of carbohydrate component into account, only the transcription level of UDP-glucose pyrophosphorylase (*UDPGP*), which could produce UDP-glucose as a substrate to synthesize carbohydrate, was detected here (Figure 4). As shown in Figure 4, the transcription level of *UDPGP* in E-81 strain was up-regulated at 72 h, although no significant difference between the two strains was found at 48 h. However, it was found that the carbohydrate content in E-81 strain was decreased, which was inconsistent with the increase in the transcription level of *UDPGP*. In previous report showed the relative transcription ratios of PKS and FAS genes affect the fatty acid component, and both the enhanced transcripts of PKS and the decreased transcripts of FAS can improve the DHA content (31, 47). Here, the transcription level ratio of *ACC* and *UDPGP* were significantly increased, which may direct more carbon resource into lipid biosynthesis (37). Therefore, it was believed that although the transcription level of *UDPGP* increases, E-81 reduces the carbohydrate synthesis efficiency through other overall regulation. Based on all of these results, it is obvious that the enhancement of fatty acid synthesis pathway could be the main reason attributed to the increased lipid production in strain E-81.

### Fed-Batch Fermentation

**Figure 5:**
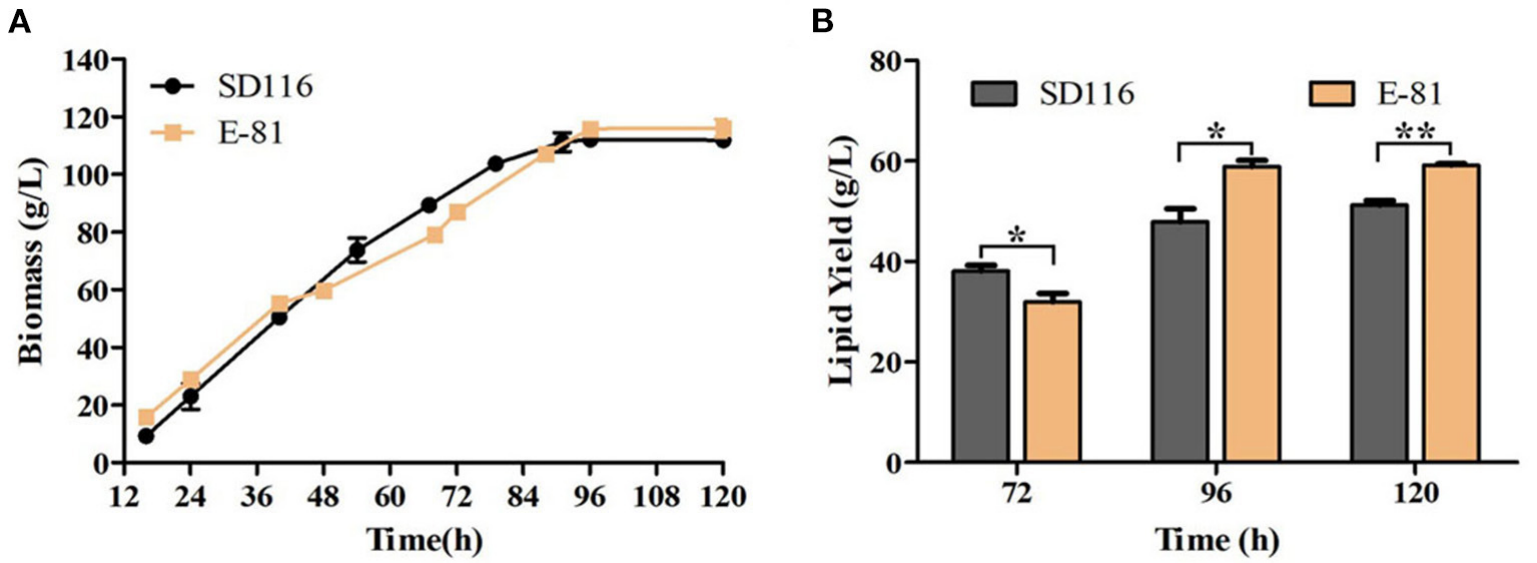
Fed-batch fermentation of SD116 and E-81 strains. **(A)** Growth curve; **(B)** total lipid yield. ^**^P < 0.01 and ^*^P < 0.05.

The fermentation performance of E-81 strain in DHA production was further investigated through a fed-batch fermentation experiment conducted with a 5 L fermentor. The final biomass of E-81 strain was 117 g/L, and no significant difference of biomass was observed between the E-81 and SD116 strains (Figure 5A). Moreover, the final glucose consumption concentration of the two strains is almost the same (315 vs. 306 g/L). However, the fatty acid composition and lipid yield had significantly changed. As shown in Figure 5B and Table 1, the DHA content was reached 52.5% of TFA, which has an increase of 33.9% compared with the original strain SD116. The lipid yields of SD116 and E-81 strain were 51.0 and 59.0 g/L, respectively, after 5 day fermentation. The total DHA yield in E-81 strain was 31.0 g/L and found a 55% increase than that of SD116 (31.0 vs. 20.0 g/L), while no significant variations in the total SFA and DPA yield were recorded.

**Table 1:** The analysis of lipid and fatty acid composition in SD116 and E-81 at the end-point of fed-batch fermentation (5th day).

**Fatty acids**	**SD116**	**E-81**	***P*-value**
C14:0 (% TFAs^*^)	2.90 ± 0.03	1.60 ± 0.60	0.036
C16:0 (% TFAs)	41.2 ± 0.46	33.9 ± 2.00	0.004
C18:0 (% TFAs)	3.01 ± 0.01	1.15 ± 0.32	0.001
ARA (% TFAs)	1.55 ± 0.01	1.17 ± 0.12	0.044
EPA (% TFAs)	1.10 ± 0.01	0.81 ± 0.09	0.030
DPA (% TFAs)	9.80 ± 0.08	8.48 ± 1.50	0.203
DHA (% TFAs)	39.2 ± 0.38	52.6 ± 2.50	0.001
Palmitic acid yield (g/L)	21.0 ± 0.6	20.0 ± 0.8	0.268
DPA yield (g/L)	5.00 ± 0.1	5.00 ± 0.6	0.847
DHA yield (g/L)	20.0 ± 0.5	31.0 ± 1.4	0.001
Total PUFAs yield (g/L)	27.0 ± 0.6	37.0 ± 1.3	0.001
Total SFA yield (g/L)	37.0 ± 0.6	37.0 ± 0.6	0.671
Total lipid yield (g/L)	51.0 ± 1.3	59.0 ± 0.4	0.001

* *TFAs, total fatty acids*.

Thraustochytrids including *Aurantiochytrium*, could use a variety of substrates as carbon sources. Generally, when glucose is used as the carbon source, the DHA content of thraustochytrids is higher, while higher lipid content can be obtained when glycerol and acetic acid are used as the carbon source, which the DHA content is slightly lower (48–50). Therefore, comprehensive considerations such as substrate cost, sustainable availability, etc. need to be considered when selecting the carbon sources. At present, fed-batch fermentation is the main method for algal based DHA production. Researchers try to improve the efficiency of the fermentation process by optimizing various parameters such as pH (49), osmotic pressure (51), and aeration methods (9). Currently, computer simulation and computer-aided have also been used to improve the fermentation process (52). Through the computer-aided design, improvements to the industrial processes could be determined without performing excessive experiments.

## Conclusions

In order to obtain non-transgenic strains with high DHA content, heavy-ion irradiation was first used to increase the genetic diversity of the original strains, and then two-step ALE: low temperature based ALE and quizalofop-p-ethyl based ALE were performed to enhance the DHA and lipid accumulation. Using this strategy, the DHA content of the end-point strain E-81 was 51% higher than that of the parent strain. This study demonstrated a non-genetic approach to enhance lipid and DHA content in non-model industrial oleaginous strains.

## Data Availability Statement

The original contributions presented in the study are included in the article/Supplementary Material, further inquiries can be directed to the corresponding author/s.

## Author Contributions

QC and XS conceived the study. SW and WW were responsible for the ALE. HZ, HL, and ZW carried out the test of fatty acids. WW and XS carried out the fermentation experiments. KA and XS performed data analysis. SW, KA, and XS wrote the paper. All the authors reviewed and approved the final manuscript.

## Funding

This work was supported by the National Key Research and Development Program (2019YFD0901904), National Natural Science Foundation of China (Nos. 42006114, 42106108, and 32001053), the Shandong Province Natural Science Foundation (ZR2020QD099), the Key Deployment Project of Centre for Ocean Mega-Research of Science, Chinese Academy of Sciences (COMS2019J07), Qingdao independent innovation major project (Grant No. 21-1-2-23-hz), and supported by QIBEBT (Grant: QIBEBT I201933). This study was also supported by Dalian National Laboratory for Clean Energy (DNL), CAS.

## Conflict of Interest

The authors declare that the research was conducted in the absence of any commercial or financial relationships that could be construed as a potential conflict of interest.

## Publisher's Note

All claims expressed in this article are solely those of the authors and do not necessarily represent those of their affiliated organizations, or those of the publisher, the editors and the reviewers. Any product that may be evaluated in this article, or claim that may be made by its manufacturer, is not guaranteed or endorsed by the publisher.

## Abbreviations

DHA: docosahexaenoic acid
ALE: adaptive laboratory evolution
PUFA: polyunsaturated fatty acids
EPA: eicosapentaenoic acid
DCW: dry cell weight
TFAs: total fatty acids
qRT-PCR: quantitative real-time PCR
LET: linear energy transfer
EMS: ethyl methane sulfonate
ACCase: acetyl-CoA carboxylase
CS: citrate synthase
ICDH: isocitrate dehydrogenase
ME: malic enzyme
PKS: polyketide synthase
UDPGP: UDP-glucose pyrophosphorylase.
