# Supplementary material for: A Two-Stage Adaptive Laboratory Evolution Strategy to Enhance Docosahexaenoic Acid Synthesis in Oleaginous Thraustochytrid
Source: Front Nutr. 2021 Dec 31;8:795491. doi: 10.3389/fnut.2021.795491 (PMC8759201; doi:10.3389/fnut.2021.795491)
Supplement: Supplementary file 1 [file Data_Sheet_1.docx]

**TABLE S1**. Primers used in this study.

| Primer | Sequence |
| --- | --- |
| CS-F | CTCTTCCCTCGATTACTCTGC |
| CS-R | CCTCGTGGTCACTGTGGAT |
| ICDH-F | CGTGACATACGATAACCCTCT |
| ICDH-R | TCTTGGTGACGACGTAGGG |
| ACC-F | GAGACCACTTACCGCCTGTT |
| ACC-R | CGCCAATGAGCACAAGGAAG |
| G6PDH-F | CCAGAGACTCCTTCGGCATAT |
| G6PDH-R | CCTCCAACACCTCGTCAAGA |
| ME-F | AAGCCTGCCAAGAGTTCCA |
| ME-R | GCCAGTTGTTCACGAGAGTC |
| FAS-F | GCATCTACCACCGTCTTGTTG |
| FAS-R | GGAGCAGAACCAGTCACCTT |
| OrfA-F | AGCCTCCTTGATAGCCTTCTC |
| OrfA-R | TCTGGTGCGTGTTCTTGGT |
| OrfB-F | GTCGGAGTAGGTGGCTTGT |
| OrfB-R | GCCTTCATCGTCACTGGTAC |
| OrfC-F | CTGGTGGTGGTGTTGGATG |
| OrfC-R | GCTGCTTGCGGACATTGT |
| UDPGP-F | GACGAAACTGGGTACTCGC |
| UDPGP-R | GGGTTCACAAACTCAGGGAT |
| Actin-F | ATCATGGTCGGCATGGAC |
| Actin-R | GTCACGATACCGTGCTCAAT |


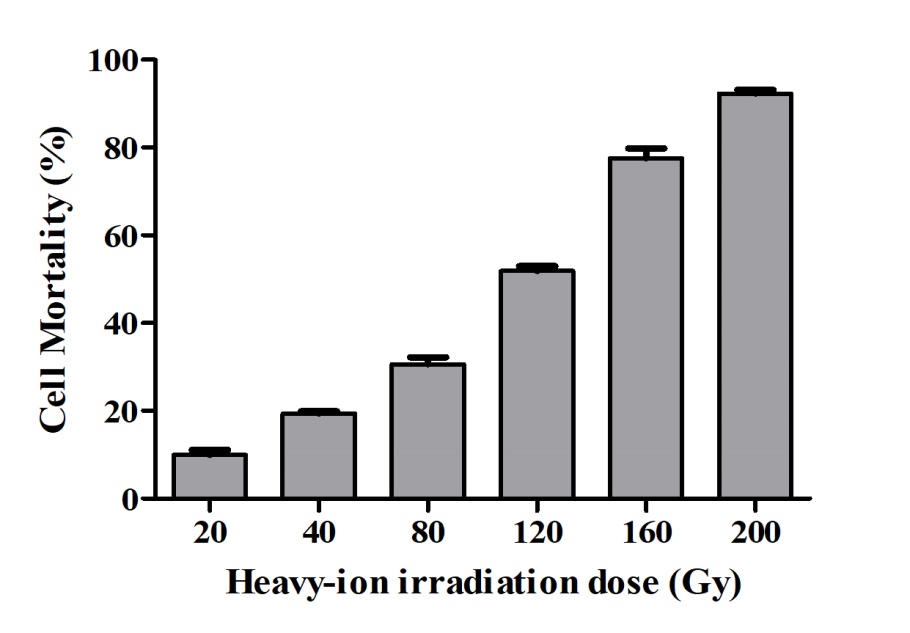


**FIGURE S1.** The mortality rate of *Aurantiochytrium* exposed to different heavy-ion irradiation doses. Values are means ± standard error (n=3).


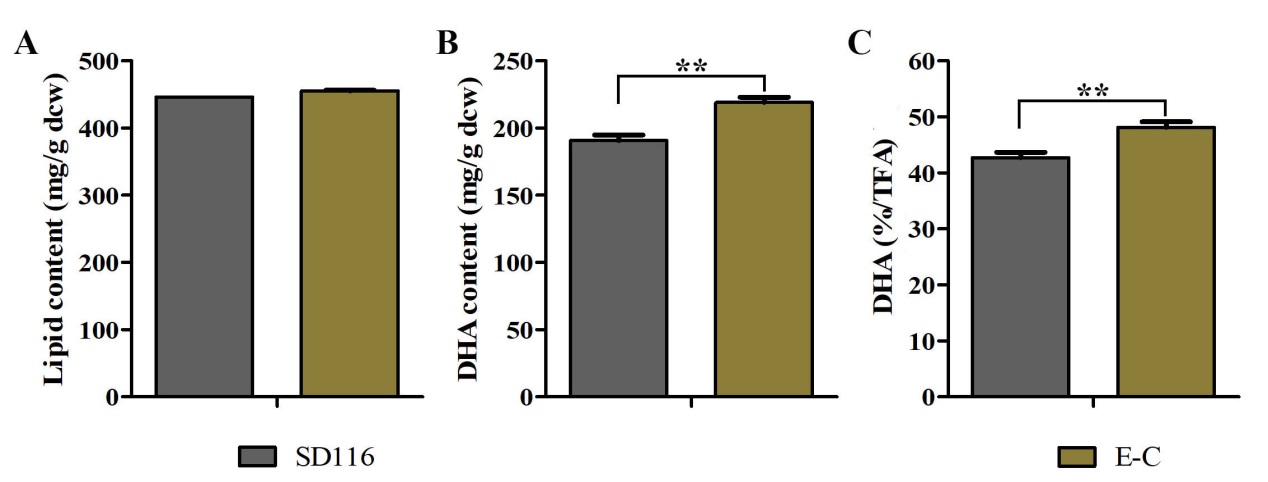


**FIGURE S2.** Lipid analysis of the evolved strains obtained from the cold stress based ALE after 96 h of fermentation. (A) Total lipids content in SD116 and E-C strains. (B) DHA content in SD116 and E-C strains. (C) DHA purity in SD116 and E-C strains. ** *P* < 0.01. * *P* < 0.05.


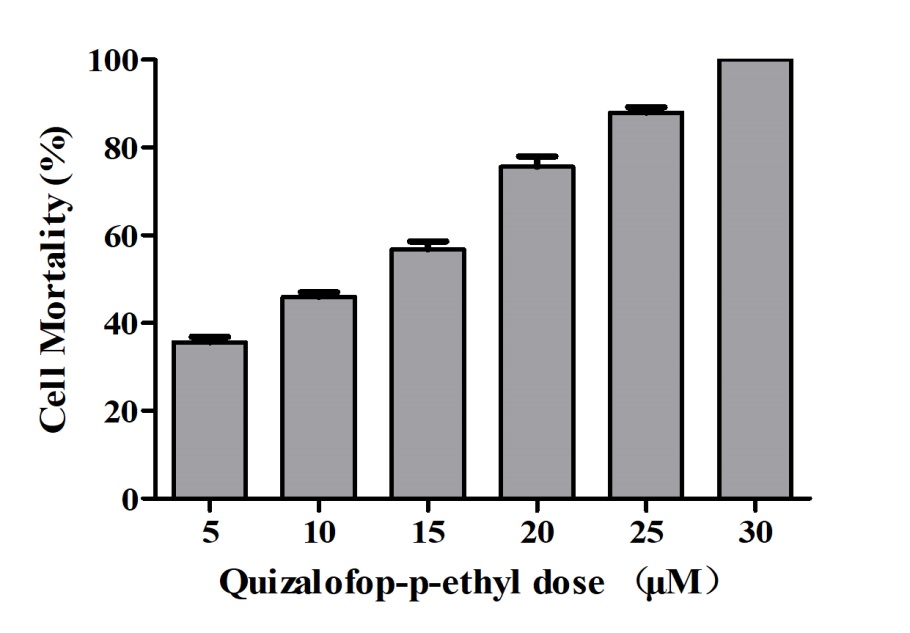


**FIGURE S3.** The cell mortality rate of *Aurantiochytrium* exposed to different concentrations of quizalofop-p-ethyl dose. Values are means ± standard errors (n=3).


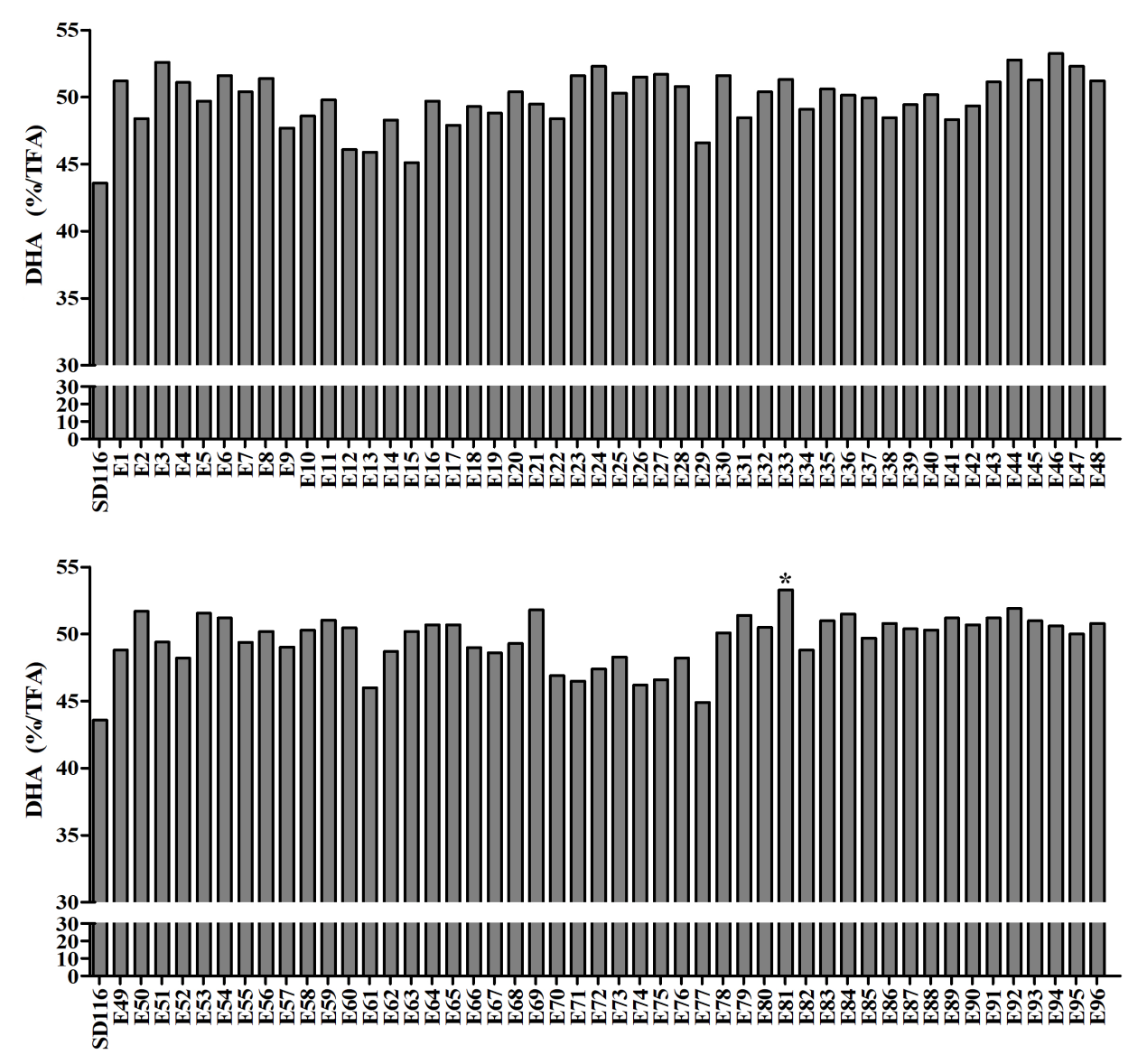


**FIGURE S4**. The DHA purity in SD116 and the evolved strains obtained from quizalofop-p-ethyl based ALE after 96 h of fermentation. * E81 strain, which has the highest DHA purity in total fatty acids.


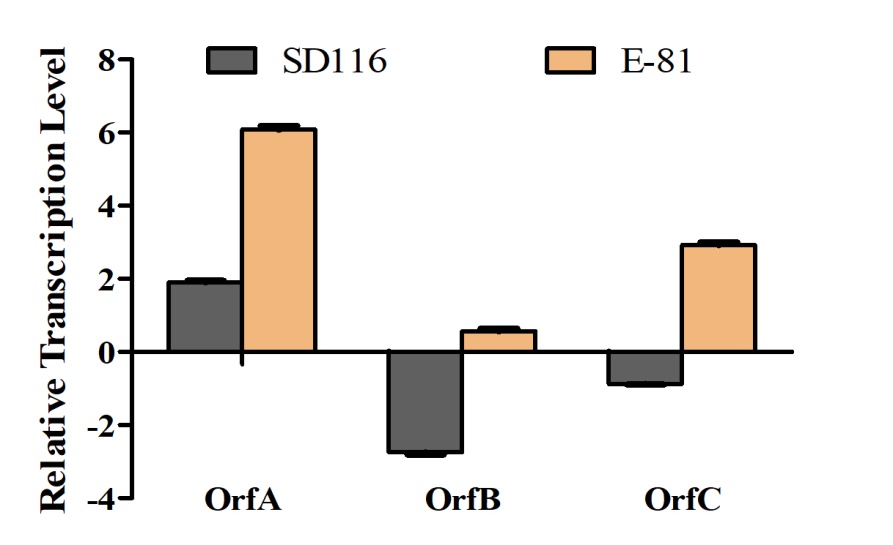


**FIGURE S5.** Relative transcription level of *orfA*, *orfB*, and *orfC* compared to that of *FAS* in SD116 and E-81 strain at 72 h.
